# Sage Tea Drinking Improves Lipid Profile and Antioxidant Defences in Humans

**DOI:** 10.3390/ijms10093937

**Published:** 2009-09-09

**Authors:** Carla M. Sá, Alice A. Ramos, Marisa F. Azevedo, Cristovao F. Lima, Manuel Fernandes-Ferreira, Cristina Pereira-Wilson

**Affiliations:** 1 CBMA–Centre of Molecular and Environmental Biology/Department of Biology, School of Sciences, University of Minho, Campus de Gualtar, 4710-057 Braga, Portugal; E-Mails:carlasa@bio.uminho.pt (C.M.S.);aliceramos@bio.uminho.pt (A.A.R.);marisazevedo@gmail.com (M.F.A.); 2 CITAB–Centre for the Research and Technology of Agro-Environment and Biological Sciences/Department of Biology, School of Sciences, University of Minho, 4710-057 Braga, Portugal; E-Mails:lima@bio.uminho.pt (C.F.L.);mfferreira@bio.uminho.pt (M.F.F.)

**Keywords:** *Salvia officinalis* L., type 2 diabetes mellitus, lipid profile, human trial, antioxidant defences

## Abstract

*Salvia officinalis* (common sage) is a plant with antidiabetic properties. A pilot trial (non-randomized crossover trial) with six healthy female volunteers (aged 40–50) was designed to evaluate the beneficial properties of sage tea consumption on blood glucose regulation, lipid profile and transaminase activity in humans. Effects of sage consumption on erythrocytes’ SOD and CAT activities and on Hsp70 expression in lymphocytes were also evaluated. Four weeks sage tea treatment had no effects on plasma glucose. An improvement in lipid profile was observed with lower plasma LDL cholesterol and total cholesterol levels as well as higher plasma HDL cholesterol levels during and two weeks after treatment. Sage tea also increased lymphocyte Hsp70 expression and erythrocyte SOD and CAT activities. No hepatotoxic effects or other adverse effects were observed.

## 1. Introduction

Diabetes mellitus is a serious public health problem characterized by deficient plasma glucose regulation due to tissue insulin resistance and/or *β*-cell failure which causes high morbidity and mortality rates. Type 2 diabetes (T2DM) accounts for the majority cases of diabetes (about 90%) and is becoming more prevalent due to the increasing rates of obesity in youth and adulthood and sedentary lifestyles [1].

Dyslipidaemia is also common among diabetic patients and plays a critical role in the development of cardiovascular complications. Metabolic dyslipidaemia is characterised by high levels of triglycerides, associated with low levels of high-density lipoprotein cholesterol–HDL-C with or without a raise in low-density lipoprotein cholesterol–LDL-C [2–5]. These imbalances in the internal metabolic environment, combined with the characteristic low antioxidant defences of diabetics can lead to oxidative stress and cellular damage. Oxidative stress has been demonstrated to be a contributor to the progression of the disease, accelerating both *β*-cell failure and cardiovascular complications. Antioxidant enzymes, such as superoxide dismutase (SOD) and catalase (CAT) play crucial roles in the cellular protection against oxidative damage eliminating reactive oxygen species (ROS) [6,7].

The increased expression of heat shock proteins (Hsp) is regarded as one of the most powerful means of cytoprotection against loss of cellular homeostasis and Hsp levels have been shown to be involved in tissue insulin responsiveness [8]. The study of levels of cell protection in the most relevant insulin sensitive tissues is highly invasive and the easily accessible lymphocyte may provide valuable biomarkers of health status [9,10]. The Hsp70 levels of lymphocytes may therefore provide information on effects on insulin response.

T2DM is preventable through lifestyles changes (including diet changes, physical exercise and weight loss) and pharmacological interventions with drugs such as metformin and acarbose [11–13]. Herbal teas with glucose-lowering properties may offer low-cost alternatives to pharmacological interventions to limit the progression of the disease while having good acceptance. In particular *Momordica charantia* has been shown to improve insulin secretion in *β*-cells, increase peripheral glucose uptake, significantly reduce serum cholesterol and triglycerides levels at the same time as increasing HDL-C levels [14]; *Coccinia indica* improves antioxidant status by increasing antioxidant defences such as SOD, CAT and reduced glutathione levels and shows a significant hypoglycaemic action by decreasing blood glucose levels and increasing hepatic glycogen synthesis in animal models [15,16] and *Camellia sinensis*, has been associated with weight reduction, decrease in blood pressure and blood glucose levels, protection against lipid peroxidation and improvement of blood lipid profile which suggest beneficial effects against obesity, cardiovascular diseases (CVD) and T2DM [17–19].

Common sage (*Salvia officinalis*) is among the plants to which antidiabetic properties have been attributed by popular medicine and its extracts showed to possess hypoglycaemic effects in normal and diabetic animals [20,21]. In a previous study we have shown that treatment with sage tea for 14 days lowered fasting plasma glucose levels but had no effects on glucose clearance in response to an intraperitoneal glucose tolerance test (ipGTT) in rats [22]. Using hepatocyte primary cultures a decreased gluconeogenesic response to glucagon and a higher responsiveness to insulin were found after *in vivo* treatment with sage tea [22]. *In vivo* treatments with *Salvia fruticosa* tea also reduced plasma glucose in STZ rats (unpublished observations).

With the purpose of studying the effects of sage tea consumption on glucose regulation in humans, a pilot trial with human volunteers was carried out where a number of parameters relevant to diabetes were analysed such as fasting and postprandial blood glucose, response to an oral glucose tolerance test–OGTT, lipid profile, liver toxicity and antioxidant defences. Demonstration that there is no toxicity or adverse effects associated with sage consumption paves the way for future studies involving diabetic patients where the true antidiabetic potential of sage will have to be tested.

## 2. Results and Discussion

### 2.1. Effects of Salvia officinalis on Blood Glucose Regulation, Plasma Aminotransferase Activity, Blood Pressure, Heart Rate at Rest and Body Weight

In this study we evaluated in healthy women volunteers the effects of *Salvia officinalis* (sage) tea drinking (300 mL, twice a day) on parameters relevant to diabetes and its associated cardiovascular complications. In spite of its claimed antidiabetic potential and traditional use, no effects on blood glucose were observed in healthy humans (Table 1). In our previous work, sage tea drinking decreased fasting blood glucose in normoglycemic mice [22]. Since no such effects on fasting blood glucose were found in the present study (Table 1), the risk of hypoglycaemia is excluded. Sage tea drinking improved lipid profile and increased antioxidant defences (see below) which may indirectly improve the diabetic condition.

Plasma aminotransferase AST and ALT activities were determined in order to evaluate the safety of *S. officinalis* tea drinking in humans. Although a significant increase in plasma AST enzyme activity was observed at the fourth week of sage tea treatment (Table 1), toxicity did not occur, since the results are well below reference values (40 IU/L) [23,24]. Thus, drinking sage tea does not cause hepatotoxicity nor does it induce other adverse effects, such as changes in blood pressure, heart rate at rest and body weight (Table 1).

In order to assess the effects of sage tea on glucose clearance, two OGTTs were performed, at baseline and at the end of sage tea treatment (four weeks after the first one), and no changes were observed (Table 2). Although all the volunteers were non-diabetic, they belong to an age group at risk of developing impaired glucose tolerance (IGT) (a pre-diabetic stage). All subjects showed no glucose intolerance. Although no effects on glucose regulation were observed in healthy humans, it remains to be established whether sage tea drinking helps to regulate blood glucose in hyperglycaemic patients.

### 2.2. Effects of Salvia officinalis on Lipid Profile

**Figure 1.**
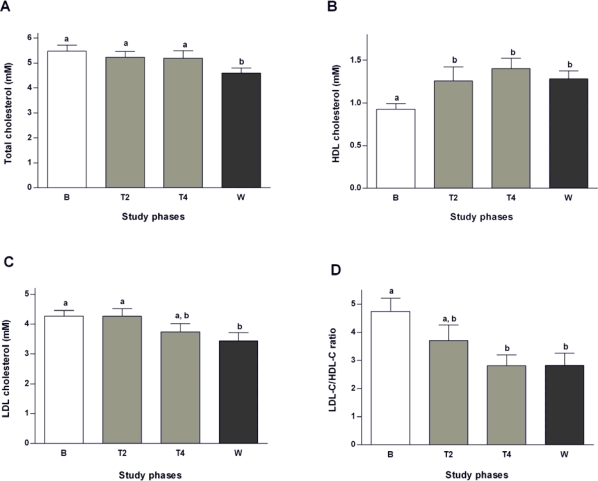
Total cholesterol [A], LDL cholesterol [B] and HDL cholesterol [C] levels as well as LDL-C/HDL-C ratio [D] in plasma measured at different points during the study: baseline (B), second (T2) and fourth week of treatment (T4), and wash-out (W). Values are mean ± SEM (n **=** 6). Groups with the same letter notation are not significantly different from each other (P > 0.05).

Sage tea treatment reduced slightly plasma total cholesterol levels during treatment phase (by 4.5% at T2 and by 5.3% at T4), achieving a significant reduction two weeks after the end of the treatment (values 16% lower than the baseline; Figure 1A). A beneficial effect on lipoprotein levels, with a gradual reduction of LDL-C (of 12.4% at the end of the treatment and 19.6% after 2 weeks wash-out; Figure 1C) and a gradual increase of HDL-C levels (50.6% at the end of the treatment and 37.6% after two weeks wash-out; Figure 1B) were observed. The LDL-C/HDL-C ratio contributes to assess the risk of cardiovascular complications due to dyslipidemia [26]. As shown in Figure 1D, sage tea gradually decreased LDL-C/HDL-C ratio from baseline until the end of four weeks of tea treatment. This ratio remained significantly reduced even after the two week wash-out period (Figure 1D). These results suggest that *S. officinalis* tea consumption is accountable for the improvement of the lipid profile inducing a decrease on the higly atherogenic LDL-C particles (which are easily oxidable and less readily cleared [27]) and an increase in the HDL-C particles, contributing, therefore, positively to the control of the dyslipidaemia frequently observed in T2DM but also related to other diseases.

A variety of pharmaceutical approaches have been developed in order to achieve both decrease of LDL-C and rise of HDL-C levels, with the aim to reduce the risk of CVD [4]. Despite the available therapies based on statins, niacin and fibrates (pharmacological agents used to lower plasma LDL-C and increase HDL-C levels), the need for more effective drugs drives the search for alternative compounds. Several natural compounds have been shown to act on cholesterol metabolism (by reducing its absorption or its synthesis), such as phytosterols and catechins [28,29]. Extracts from some sage species have been shown to be effective in the prevention of cardiovascular diseases due to, at least in part, prevention of LDL-C oxidation [30]. Sage tea drinking had no significant effects on post-prandial triglycerides (data not shown).

### 2.3. Effects of Salvia officinalis on Antioxidant Defences and Heat-shock Protein 70 (Hsp70) Expression

**Figure 2.**
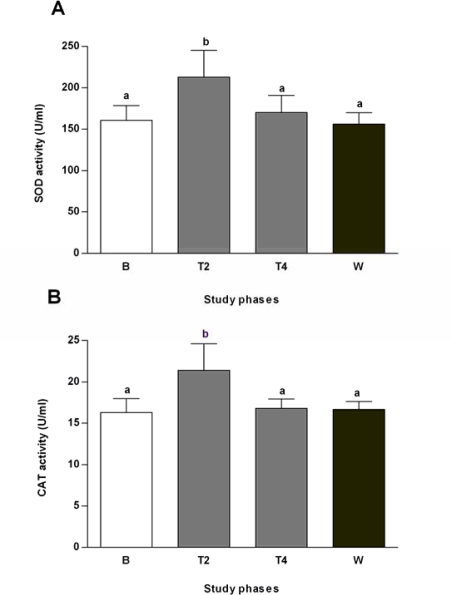
Antioxidant activities of SOD [A] and CAT [B] measured in haemolysed erythrocytes. Samples were taken at different time points during the study: baseline (B), second (T2) and fourth week of treatment (T4) and wash-out (W). Values are mean ± SEM (n = 6). Groups with the same letter notation are not significantly different from each other (P > 0.05).

Sage tea drinking improved human erythrocyte antioxidant status by significantly increasing SOD and CAT activities after two weeks of sage treatment, returning afterwards to normal values (Figure 2A and 2B). The antioxidant properties of sage tea, in addition to preventing lipoprotein oxidation, may also protect cells from diabetes’ related gluco- and lipotoxicity and prevent progressive *β*-cell destruction, which could provide long term protection of these insulin-producing cells.

The antioxidant activity of phenolic compounds has been widely studied and it is known that these compounds can either stimulate endogenous antioxidant defence systems or scavenge reactive species [31]. Rosmarinic acid and luteolin-7-glucoside are the two most representative phenolic compounds present in our *S. officinalis* extracts (tea) [32]. These phenolic compounds showed protective effects against oxidative damage in hepatocytes, and limited GSH depletion induced by *tert*-butyl hydroperoxyde in HepG2, preserving cell viability [33]. The same happened for sage extracts in HepG2 cells [34]. In animals, sage tea drinking also stimulated several antioxidant enzymes in the liver [32,35], corroborating the effects of this tea in human erythrocytes observed in the present study.

**Figure 3.**
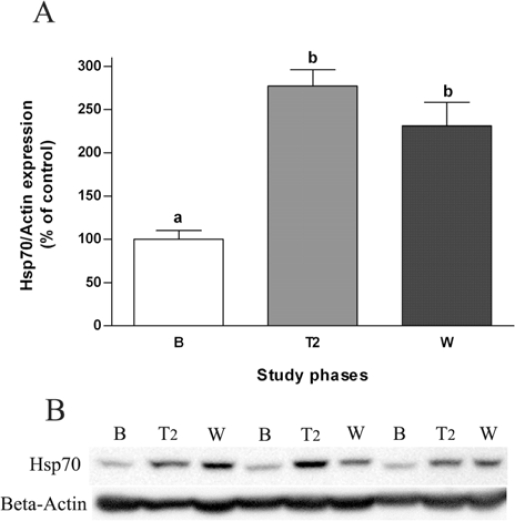
Western blot analysis of the inducible form of Hsp70 (Hsp72) in human lymphocytes at the different points during the study: baseline (B), second week of treatment (T2) and wash-out (W). [A] Values are mean ± SEM (n = 6). Groups with the same letter notation are not significantly different from each other (P > 0.05). [B] Representative immunoblots of three subjects (*β*-actin was used as loading control).

Since lymphocytes may provide valuable and easily accessible biomarkers of the health status of individuals [9,10] and heat shock proteins have been involved in tissue insulin responsiveness [8], the expression of Hsp70 in human lymphocyte lysates was evaluated. The lymphocyte’s inducible Hsp72 protein not only significantly increased at the second week of *S. officinalis* tea treatment (about 2.8-fold) but also remained elevated in the wash-out period (Figure 3). These findings suggest a beneficial potential of sage tea drinking on Hsp72 protein induction, an endogenous stress modulator, which plays a crucial role in cellular homeostasis decreasing the risk of development of T2DM by blocking inflammatory signalling molecules including c-Jun amino terminal kinase (JNK), inhibitor of kB kinase (IKK) and tumor necrosis factor-α (TNF-α) in insulin responsive tissues [36–39]. These molecules phosphorylate insulin receptor substrate-1 (IRS-1) in specific serine sites and determine decreased insulin sensitivity. Indeed, Hsp72 gene and protein expression has been shown to be significantly reduced in T2DM patients and correlated with reduced insulin sensitivity [40–42]. The antioxidant alpha-lipoic acid showed recently to improve insulin action in high-fat-fed rats by increasing the expression of Hsp72 and consequently inhibiting JNK and IKK [43]. Therefore, an increase in inducible Hsp70 protein expression by sage tea would represent an amelioration of whole-body insulin sensitivity although the assumption that lymphocyte Hsp levels mimic other tissues Hsp levels requires further demonstration.

Heat shock proteins confer also cytoprotection and assure survival after environmental stresses, being therefore implicated in infection, immunity and aging, as well as in ischemic and neurodegenerative diseases [44]. Thus, induction of Hsp72 by sage tea could also be useful by conferring stress tolerance and cytoprotection against several environmental-induced injury conditions helping increase lifespan and prevente age-related diseases such as diabetes, cancer and neurodegeneration. Natural compounds such as resveratrol have been shown recently to induce the heat-shock response and to protect human cells from severe heat stress [44]. As well, paeoniflorin isolated from *Paeonia lactifora* showed to induce heat shock proteins expression and to afford termotolerance in cultured cells [45].

## 3. Experimental Section

### 3.1. Subjects and Study Design

**Figure 4.**
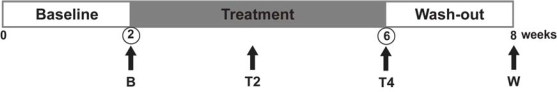
Experimental outline of the pilot study. Blood samples were taken at the times indicated by the arrows. Oral glucose tolerance test were performed at the times indicated by the circles.

Six healthy female volunteers (aged 40–50) participated in this trial after signing an informed consent form. The whole study was carried out in accordance with the principles of the Declaration of Helsinki. Smokers and subjects on regular medication were excluded from the study. Effects of sage tea drinking on body weight, blood pressure and heart rate at rest were recorded at first week of baseline and the end of each of the eight weeks of the trial. Weekly records of perceived negative events and concomitant medication were also kept. All the volunteers completed the study and reported no side effects. A non-randomized crossover study, where individuals serve as their own controls, was carried out in three phases: two weeks of baseline, four weeks of sage tea treatment and two weeks of wash-out (Figure 4). The two-week baseline phase was included in order to obtain control values for all the volunteers. During this phase, all the parameters were measured and values are presented in figures and tables as basal levels. A treatment phase with sage tea followed, where 300 mL of tea were taken twice daily for four weeks. Sampling was carried out at the end of second and fourth week of sage treatment. A two-week wash-out phase was included after treatment with the aim to assess the duration of sage tea effects beyond the treatment period.

### 3.2. Plant Material and Preparation of S. officinalis Tea

*Salvia officinalis L.* plants were grown in an experimental farm located in Arouca, Portugal, and were collected in April, 2001. The aerial parts of plants were lyophilized and kept at −20 °C. The sage tea was routinely prepared by pouring 300 mL of boiling water onto 4 g dried plant material and allowing to steep for 5 min [32]. This infusion yielded about 3.5 ± 0.1 mg lyophilized extract dry weight per mL, where rosmarinic acid (362 mg/mL infusion) and luteolin-7-glucoside (115.3 mg/mL infusion) were the major phenolic compounds, and 1,8-cineole, *cis*-thujone, *trans*-thujone, camphor and borneol the major volatile components (4.8 mg/mL infusion). For full extract characterization see [32].

### 3.3. Blood Samples, Erythrocytes’ Hemolysates and Lymphocytes Lysates

At the different sampling points (baseline–B, second week of treatment–T2, fourth week of treatment–T4 and at the end of wash-out–W), venous blood samples were collected postprandially in EDTA vacutainers (Vacuett®, Greiner Bio-one GmbH, Austria). An aliquot of blood was used for measuring glucose levels. Immediately after sampling, about 3 mL of blood were centrifuged at 200× g (KUBOTA 2100, Tokyo, Japan) for 10 min to separate the plasma. Plasma aliquots were stored at −80 °C for later measurements of transaminases, total cholesterol, HDL-C and LDL-C levels. The remaining erythrocyte enriched fraction was haemolysed to analyse SOD and CAT activity. About 10 mL of blood were used to separate peripheral blood lymphocytes (PBLs) by a Ficoll density gradient centrifugation following the procedure provided by the Ficoll manufacturer (Ficoll Paque-Plus, GE Healthcare, Piscataway, NJ, USA). The resultant PBL fraction was collected, washed with PBS and the cell pellet was homogenised with lysis buffer (25 mM KH_2_PO_4_, pH 7.5, 2 mM MgCl_2_, 5 mM KCl, 1 mM EDTA, 1 mM EGTA, with 0.1 mM PMSF and 2 mM DTT added fresh). Protein concentration from lymphocyte lysates was measured with the Bradford reagent (Sigma-Aldrich, Inc., St. Louis, MO, USA) and aliquots kept at −80 °C for later quantification of Hsp70.

### 3.4. Measurement of Blood and Plasma Parameters

#### 3.4.1. Quantification of Glucose Levels and Oral Glucose Tolerance Test (OGTT)

Fasting and postprandial glycaemia were measured with the Accutrend® GCT device (Roche Diagnostics GmbH, Mannheim, Germany) using Accutrend® test strips for glucose (Roche Diagnostics GmbH). Two OGTTs were performed after an overnight fast one at baseline and the other at week four of sage tea treatment. For that, 1 g of glucose per Kg body weight of each volunteer was given in up to 300 mL of warm water, which was consumed within 5 min of start. The OGTT started when the subjects began drinking with blood sampling taken before as well as at 45 min and 165 min after the oral glucose load. Blood glucose concentration was measured as above.

#### 3.4.2. Characterization of Lipid Profile

Total plasma cholesterol, LDL-C and HDL-C levels were measured in plasma using spectrophotometric commercial kits from Spinreact (Girona, Spain), according to the manufacturer’s specifications.

#### 3.4.3. Quantification of Plasma Aminotransferases

The alanine aminotransferase (ALT) and aspartate aminotransferase (AST) activities were measured spectrophotometrically in plasma following the NADH oxidation method at 340 nm on a plate reader (Spectra Max 340 pc, Molecular Devices, Sunnyvale, CA, USA), as previously described [32].

#### 3.4.4. Quantification of Erythrocytes’ Antioxidant Defences

The haemolysate fraction was used to determine SOD activity using the Ransod kit (Randox, Crumlin, UK) following the manufacturer’s specifications. The SOD activity in haemolysates was expressed as U/mL, with 1U corresponding to 50% of inhibition of 2-(4-iodophenyl)-3-(4-nitro-phenol)-5-phenyltetrazolium chloride (INT) reduction under assay conditions. The same haemolysates were used to measure CAT activity as described elsewhere [46]. In brief, the decomposition of H_2_O_2_ was followed at 240 nm in a spectrophotometer (Cary IE, UV-Visible Spectrophotometer Varian, Australia) and the activity expressed as U/mL (U being μmol of H_2_O_2_ decomposed per minute) using the molar extinction coefficient of 0.0394 mL μmol^–1^ cm^–1^.

#### 3.4.5. Western Blot Analyses

The quantification of Hsp70 protein in lymphocyte lysates was assessed by Western Blot in which proteins (20 μg per sample) were separated by SDS-PAGE using the mini-PROTEAN 3 electrophoresis cell (BioRad Laboratories, Inc., Hercules, CA, USA). Proteins were then transferred onto Hybond-P polyvinylidene difluoride membrane (GE Healthcare, UK) and membranes blocked in 5% (w/v) non-fat dry milk in TPBS (0.05% (v/v) Tween 20 in PBS, pH 7.4). Blotted membranes were probed with mouse monoclonal antibodies against Hsp72 (StressGen, Assay Designs, Inc., Ann Arbor, MI, USA) and *β*-actin (Sigma; used as loading control). Bound antibodies were then detected by chemiluminescence using appropriate secondary antibodies and the reactive bands acquired with a ChemiDoc XRS (BioRad) imaging densitometer. Band intensity was quantified using the Quantity One image analysis software (BioRad).

### 3.5. Statistical Analysis

Data are expressed as means ± SEM (n = 6). For statistical analysis the different parameters were analysed by repeated one-way ANOVA measurements followed by the Student-Newman-Keuls post-test (GraphPad Prism, version 4.03; GraphPad Software Inc., San Diego, CA, USA) to identify differences between studied time points. P values ≤ 0.05 were considered statistically significant (with a confidence interval of 95%).

## 4. Conclusions

In conclusion, a four week treatment with sage tea was effective in the improvement of lipid profile, antioxidant defences and lymphocyte Hsp70 protein expression of human volunteers, which in the long term may be responsible for the general health improving properties attributed to sage. Our results support the popular believe that *S. officinalis* tea is beneficial and although not demonstrating effects on glucose regulation in healthy individuals, they show that sage tea drinking is safe and pave the way for sage’s effects to be tested in diabetic patients.

## Figures and Tables

**Table 1.** Physiological and biochemical parameters during the different phases of the trial: baseline (B), second (T2) and fourth (T4) week of sage tea treatment and wash-out (W).

**Parameters**	**Phases of the trial**			
	**B**	**T2**	**T4**	**W**
Weight (kg)	56.2 ± 7.1	56.2 ± 6.1	55.6 ± 6.1	55.9 ± 5.9
Systolic blood pressure (mmHg)	116.1 ± 10.3	110.3 ± 14.5	110.7 ± 15.5	107.7 ± 13.2
Diastolic blood pressure (mmHg)	68.2 ± 9.4	64.5 ± 14.0	63.6 ± 11.7	59.5 ± 9.1
Heart rate at rest (beats/min)	65.9 ± 10.7	67.2 ± 10.9	66.6 ± 8.2	68.7 ± 9.9

ALT (IU/L)	7.3 ± 1.0	6.8 ± 1.4	8.4 ± 1.6	7.6 ± 1.5
AST (IU/L)	8.1 ± 1.1	10.0 ± 2.0	10.6 ± 1.8*	9.8 ± 1.2
Fasting glucose levels^a^ (mM)	4.31 ± 1.18	4.60 ± 0.92	4.21 ± 1.54	---
Postprandial glucose levels^a^ (mM)	5.33 ± 1.64	4.35 ± 0.53	4.88 ± 0.94	4.58 ± 0.90

Values are mean ± SEM (n = 6).

* *P* ≤ 0.05 when compared with baseline values.

^a^ Glucose concentration in blood.

**Table 2.** Blood glucose concentration in response to an oral glucose tolerance test (OGTT).

	**Blood glucose levels (mM)**		
**Time**	**0 min**	**45 min**	**165 min**
**Baseline**	4.3 ± 1.2	5.5 ± 1.2	4.3 ± 0.4
**Treatment (4 weeks)**	4.2 ± 1.5	6.9 ± 1.4	4.5 ± 0.9

Values are mean ± SEM (n = 6). The reference values for a non-diabetic individual to the standard OGTT (75 g glucose/300 mL water) are: 3.33–5.56 mM (before glucose loading); < 10 mM (0.5–1.5 h after glucose loading) and 3.33–5.56 mM (3 h after glucose loading) [25].

## References

[1] Williams G Pickup JC Handbook of Diabetes3rd ed Blackwell Publishing Ltd Oxford, UK 2004 55 71

[2] Moller DE New drug targets for type 2 diabetes and the metabolic syndrome Nature 2001 414 821 827 11742415 10.1038/414821a

[3] Saxena R Madhu SV Shukla R Prabhu KM Gambhir JK Postprandial hypertriglyceridemia and oxidative stress in patients of type 2 diabetes mellitus with macrovascular complications Clin. Chim. Acta 2005 359 101 108 15893742 10.1016/j.cccn.2005.03.036

[4] Toth PP High-density lipoprotein as a therapeutic target: Clinical evidence and treatment strategies Am. J. Cardiol 2005 96 50K 58K 10.1016/j.amjcard.2005.08.008 16291015

[5] Veiraiah A Hyperglycemia, lipoprotein glycation, and vascular disease Angiology 2005 56 431 438 16079928 10.1177/000331970505600411

[6] Celik I Isik I Determination of chemopreventive role of *Foeniculum vulgare* and *Salvia officinalis* infusion on trichloroacetic acid-induced increased serum marker enzymes lipid peroxidation and antioxidative defense systems in rats Nat. Prod. Res 2008 22 66 75 17999340 10.1080/14786410701590426

[7] Matés JM Pérez-Gómez C Castro IN Antioxidant enzymes and human diseases Clin. Biochem 1999 32 595 603 10638941 10.1016/s0009-9120(99)00075-2

[8] Feder ME Hofmann GE Heat-shock proteins, molecular chaperones, and the stress response: Evolutionary and ecological physiology Annu. Rev. Physiol 1999 61 243 282 10099689 10.1146/annurev.physiol.61.1.243

[9] Bonassi S Au WW Biomarkers in molecular epidemiology studies for health risk prediction Mutat. Res. 2002 511 73 86 11906843 10.1016/s1383-5742(02)00003-0

[10] Jin X Wang R Xiao C Cheng L Wang F Yang L Feng T Chen M Chen S Fu X Deng J Wang R Tang F Wei Q Tanguay RM Wu T Serum and lymphocyte levels of heat shock protein 70 in aging: A study in the normal Chinese population Cell Stress Chaperon 2004 9 69 75 10.1379/477.1 PMC1065308 15270079

[11] Costacou T Mayer-Davis EJ Nutrition and prevention of type 2 diabetes Annu. Rev. Nutr. 2003 23 147 170 12626686 10.1146/annurev.nutr.23.011702.073027

[12] Gruber A Nasser K Smith R Sharma JC Thomson GA Diabetes prevention: Is there more to it than lifestyle changes? Int. J. Clin. Pract 2006 60 590 594 16700860 10.1111/j.1368-5031.2006.00929.x

[13] Jermendy G Can type 2 diabetes mellitus be considered preventable? Diabetes Res. Clin. Pract 2005 68 S73 S81 15955380 10.1016/j.diabres.2005.03.010

[14] Fernandes NP Lagishetty CV Panda VS Naik SR An experimental evaluation of the antidiabetic and antilipidemic properties of a standardized *Momordica charantia* fruit extract BMC Complement Altern. Med. 2007 24 7 29 10.1186/1472-6882-7-29 PMC2048984 17892543

[15] Kumar GP Sudheesh S Vijayalakshmi NR Hypoglycaemic effect of *Coccinia indica*: Mechanism of action Planta Med 1993 59 330 332 8372150 10.1055/s-2006-959693

[16] Venkateswaran S Pari L Effect of *Coccinia indica* leaves on antioxidant status in streptozotocin-induced diabetic rats J. Ethnopharmacol 2003 84 163 168 12648810 10.1016/s0378-8741(02)00294-5

[17] Coimbra S Santos-Silva A Rocha-Pereira P Rocha S Castro E Green tea consumption improves plasma lipid profile in adults Nutr. Res 2006 26 604 607

[18] Coimbra S Castro E Rocha-Pereira P Rebelo I Rocha S Santos-Silva A The effect of green tea in oxidative stress Clin. Nutr 2006 25 790 796 16698148 10.1016/j.clnu.2006.01.022

[19] Polychronopoulos E Zeimbekis A Kastorini CM Papairakleous N Vlachou I Bountziouka V Panagiotakos DB Effects of black and green tea consumption on blood glucose levels in non-obese elderly men and women from Mediterranean Islands (MEDIS epidemiological study) Eur. J. Nutr 2008 47 10 16 18204918 10.1007/s00394-007-0690-7

[20] Alarcon-Aguilar FJ Roman-Ramos R Flores-Saenz JL Aguirre-Garcia F Investigation on the hypoglycaemic effects of extracts of four Mexican medicinal plants in normal and alloxan-diabetic mice Phytother. Res 2002 16 383 386 12112298 10.1002/ptr.914

[21] Eidi M Eidi A Zamanizadeh H Effect of *Salvia officinalis* L. leaves on serum glucose and insulin in healthy and streptozotocin-induced diabetic rats J. Ethnopharmacol 2005 100 310 313 16125023 10.1016/j.jep.2005.03.008

[22] Lima CF Azevedo MF Araujo R Fernandes-Ferreira M Pereira-Wilson C Metformin-like effects of *Salvia officinalis* (common sage): Is it useful in diabetes prevention? Br. J. Nutr. 2006 96 326 333 16923227 10.1079/bjn20061832

[23] Jamal MM Soni A Quinn PG Wheeler DE Arora S Johnston DE Clinical features of hepatitis C-infected patients with persistently normal alanine transaminase levels in the Southwestern United States Hepatology 1999 30 1307 1311 10534355 10.1002/hep.510300526

[24] Kim HC Nam CM Jee SH Han KH Oh DK Suh I Normal serum aminotransferase concentration and risk of mortality from liver diseases: Prospective cohort study BMJ 2004 328 983 986 15028636 10.1136/bmj.38050.593634.63 PMC404493

[25] Ravel R Tests for diabetes and hypoglycaemica Clinical Laboratory Medicine: Clinical Application of Laboratory Data5th ed Mosby-Year Book Inc St. Louis, MO, USA 1989Chapter 27, 463 466

[26] Sullivan DR Screening for cardiovascular disease with cholesterol Clin. Chim. Acta 2002 315 49 60 11728410 10.1016/s0009-8981(01)00720-3

[27] Nesto RW Beyond low-density lipoprotein: Addressing the atherogenic lipid triad in type 2 diabetes mellitus and the metabolic syndrome Am. J. Cardiovasc. Drugs 2005 5 379 387 16259526 10.2165/00129784-200505060-00005

[28] Plana N Nicolle C Ferre R Camps J Cós R Villoria J Masana L DANACOL group Plant sterol-enriched fermented milk enhances the attainment of LDL-cholesterol goal in hypercholesterolemic subjects Eur. J. Nutr 2008 47 32 39 18193377 10.1007/s00394-007-0693-4

[29] Raederstorff DG Schlachter MF Elste V Weber P Effect of EGCG on lipid absorption and plasma lipid levels in rats J. Nutr. Biochem 2003 14 326 332 12873714 10.1016/s0955-2863(03)00054-8

[30] Chen YL Yang SP Shiao MS Chen JW Lin SJ *Salvia miltiorrhiza* inhibits intimal hyperplasia and monocyte chemotactic protein-1 expression after balloon injury in cholesterol-fed rabbits J. Cell Biochem 2001 83 484 493 11596116 10.1002/jcb.1233

[31] Scalbert A Manach C Morand C Rémésy C Jiménez L Dietary polyphenols and the prevention of diseases Crit. Rev. Food Sci. Nutr 2005 45 287 306 16047496 10.1080/1040869059096

[32] Lima CF Andrade PB Seabra RM Fernandes-Ferreira M Pereira-Wilson C The drinking of a *Salvia officinalis* infusion improves liver antioxidant status in mice and rats J. Ethnopharmacol 2005 97 383 389 15707779 10.1016/j.jep.2004.11.029

[33] Lima CF Fernandes-Ferreira M Pereira-Wilson C Phenolic compounds protect HepG2 cells from oxidative damage: Relevance of glutathione levels Life Sci 2006 79 2056 2068 16857214 10.1016/j.lfs.2006.06.042

[34] Lima CF Valentao PC Andrade PB Seabra RM Fernandes-Ferreira M Pereira-Wilson C Water and methanolic extracts of *Salvia officinalis* protect HepG2 cells from t-BHP induced oxidative damage Chem. Biol. Interact 2007 167 107 115 17349617 10.1016/j.cbi.2007.01.020

[35] Lima CF Fernandes-Ferreira M Pereira-Wilson C Drinking of *Salvia officinalis* tea increases CCl(4)-induced hepatotoxicity in mice Food Chem. Toxicol 2007 45 456 464 17084954 10.1016/j.fct.2006.09.009

[36] Gabai VL Meriin AB Mosser DD Caron AW Rits S Shifrin VI Sherman MY Hsp70 prevents activation of stress kinases. A novel pathway of cellular thermotolerance J. Biol. Chem 1997 272 18033 18037 9218432 10.1074/jbc.272.29.18033

[37] McCarty MF Induction of heat shock proteins may combat insulin resistance Med. Hypotheses 2006 66 527 534 16309849 10.1016/j.mehy.2004.08.033

[38] Meldrum KK Burnett AL Meng X Misseri R Shaw MB Gearhart JP Meldrum DR Liposomal delivery of heat shock protein 72 into renal tubular cells blocks nuclear factor-kappaB activation, tumor necrosis factor-alpha production, and subsequent ischemia-induced apoptosis Circ. Res. 2003 92 293 299 12595341 10.1161/01.res.0000057754.35180.99

[39] Park HS Lee JS Huh SH Seo JS Choi EJ Hsp72 functions as a natural inhibitory protein of c-Jun N-terminal kinase EMBO J 2001 20 446 456 11157751 10.1093/emboj/20.3.446 PMC133486

[40] Bruce CR Carey AL Hawley JA Febbraio MA Intramuscular heat shock protein 72 and heme oxygenase-1 mRNA are reduced in patients with type 2 diabetes: Evidence that insulin resistance is associated with a disturbed antioxidant defense mechanism Diabetes 2003 52 2338 2345 12941774 10.2337/diabetes.52.9.2338

[41] Kurucz I Morva A Vaag A Eriksson KF Huang X Groop L Koranyi L Decreased expression of heat shock protein 72 in skeletal muscle of patients with type 2 diabetes correlates with insulin resistance Diabetes 2002 51 1102 1109 11916932 10.2337/diabetes.51.4.1102

[42] Chung J Nguyen AK Henstridge DC Holmes AG Chan MH Mesa JL Lancaster GI Southgate RJ Bruce CR Duffy SJ Horvath I Mestril R Watt MJ Hooper PL Kingwell BA Vigh L Hevener A Febbraio MA HSP72 protects against obesity-induced insulin resistance Proc. Natl. Acad. Sci. USA 2008 105 1739 1744 18223156 10.1073/pnas.0705799105 PMC2234214

[43] Gupte AA Bomhoff GL Morris JK Gorres BK Geiger PC Lipoic acid increases heat shock protein expression and inhibits stress kinase activation to improve insulin signaling in skeletal muscle from high-fat-fed rats J. Appl. Physiol 2009 106 1425 1434 19179648 10.1152/japplphysiol.91210.2008

[44] Putics A Végh EM Csermely P Soti C Resveratrol induces the heat-shock response and protects human cells from severe heat stress Antioxid. Redox. Signal 2008 10 65 75 17956190 10.1089/ars.2007.1866

[45] Yan D Saito K Ohmi Y Fujie N Ohtsuka K Paeoniflorin, a novel heat shock protein-inducing compound Cell Stress Chaperones 2004 9 378 389 15633296 10.1379/CSC-51R.1 PMC1065277

[46] Aebi H Catalase *in vitro* Methods Enzymol 1984 105 121 126 6727660 10.1016/s0076-6879(84)05016-3

